# Live imaging YAP signalling in mouse embryo development

**DOI:** 10.1098/rsob.210335

**Published:** 2022-01-19

**Authors:** Bin Gu, Brian Bradshaw, Min Zhu, Yu Sun, Sevan Hopyan, Janet Rossant

**Affiliations:** ^1^ Program in Developmental and Stem Cell Biology, Hospital for Sick Children, Toronto, Ontario, Canada M5G 0A4; ^2^ Department of Molecular Genetics, University of Toronto, Toronto, Ontario, Canada M5S 1A8; ^3^ Department of Mechanical and Industrial Engineering, University of Toronto, Toronto, Ontario, Canada M5S 3G8; ^4^ Institute of Biomaterials and Biomedical Engineering, University of Toronto, Toronto, Ontario, Canada M5S 3G9; ^5^ Department of Electrical and Computer Engineering, University of Toronto, Toronto, Ontario, Canada M5S 3G4; ^6^ Division of Orthopaedic Surgery, The Hospital for Sick Children, University of Toronto, Toronto, Ontario, Canada M5G 1X8

**Keywords:** mouse embryo development, HIPPO-YAP singling, live imaging, knock-in reporter

## Abstract

YAP protein is a critical regulator of mammalian embryonic development. By generating a near-infrared fusion YAP reporter mouse line, we have achieved high-resolution live imaging of YAP localization during mouse embryonic development. We have validated the reporter by demonstrating its predicted responses to blocking LATS kinase activity or blocking cell polarity. By time lapse imaging preimplantation embryos, we revealed a mitotic reset behaviour of YAP nuclear localization. We also demonstrated deep tissue live imaging in post-implantation embryos and revealed an intriguing nuclear YAP pattern in migrating cells. The YAP fusion reporter mice and imaging methods will open new opportunities for understanding dynamic YAP signalling *in vivo* in many different situations.

## Graphical abstract

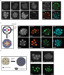

## 1. Background

*Yes-associated protein 1 (Yap1)*—commonly referred to as YAP—is a signalling protein that serves as a hub of biochemical and mechanical sensing [1–3]. YAP plays crucial regulatory roles in enormously diverse processes in development and disease, from the very beginning of life, such as during mammalian preimplantation development [4], to the very late stage of disease, such as in cancer metastasis [5]. Real-time tracking of the dynamic YAP signalling status *in vivo* would offer crucial insights into the fundamental regulatory mechanisms of mammalian development and disease.

YAP is a transcriptional co-activator for the TEA domain (TEAD) family transcription factors [6]. In response to numerous signals, YAP shifts its sub-cellular distribution to the nucleus where it associates with TEAD proteins to activate gene expression. Thus, monitoring the nuclear/cytoplasmic localization of YAP is the most broadly applied method to evaluate YAP activity [6,7]. Currently, this is primarily achieved by immunostaining of fixed embryos or tissue sections, precluding the acquisition of dynamic information. Although live imaging has been achieved in Drosophila and human cell lines and revealed intriguing YAP behaviours [8,9], no appropriate tools exist to date for live imaging endogenous YAP protein in mammals *in vivo*. Here, we report a YAP fusion reporter mouse line that allows *in vivo* live imaging of YAP behaviour.

## 2. Methods

### 2.1. Designs of guide RNAs and knock-in repair donors

A guide RNA target spanning the Stop TAG codon: TCACGTGGTTATAGAGCTGCAGG was selected using the CRISPOR algorithm (http://crispor.tefor.net) based on specificity scores [10]. The chemically modified single guide RNA (sgRNA) with the sequence of UUGCGCGGGCUCCAUGGCUG was synthesized by Synthego Inc. The repair donor for YAP-emiRFP670 reporter was designed as illustrated in Extended figure 1*a* in the electronic supplementary material. The emiRFP670 and the linker coding sequences in proper orientation were flanked by long homology arms (813 bp 5′ arm and 717 bp 3′arm) on each side and replaced the stop codon of the *Yap1* gene. The donor DNA sequence was synthesized by Epoch Life Science (http://www.epochlifescience.com) and cloned into a PBSK plasmid backbone.

**Figure 1.**
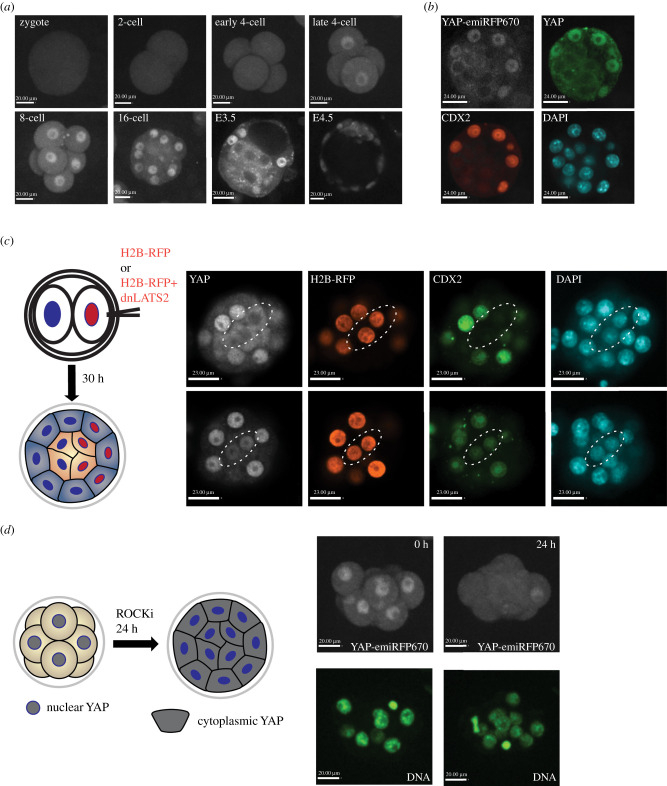
Characterization and validation of YAP-emiRFP670 reporter in preimplantation embryos: (*a*) Live imaging of YAP-emiRFP670 localization in preimplantation embryos. (*b*) Immunofluorescence images of the YAP-emiRFP670, endogenous YAP protein (Immunofluorescence) and CDX2 protein (Immunofluorescence) in a mouse blastocyst, showing perfect colocalization of YAP and CDX2 in the embryo. (*c*) Manipulation of YAP-emiRFP670 localization by expressing a dominant-negative LATS2. Left: A schematic for mRNA injection experiment. mRNAs of H2B-RFP (control group) or H2B-RFP + dnLATS2(experimental group) were injected into one of the two cells of a 2-cell stage mouse embryo and then cultured to early blastocyst stage for analysis. Right upper panel, a control embryo shows that the expression of H2B-RFP did not cause nuclear YAP localization and CDX2 expression. Right bottom panel, a dnLATS2 injected embryo showed nuclear YAP and CDX2 expression in inside cells. Cells of interest were circled by dotted lines. (*d*) Manipulation of YAP-emiRFP670 localization by treating with the ROCK inhibitor Y27632. Left: A schematic for the treatment experiment. Right, Snapshots from live imaging series electronic supplementary material, movie S1 showing ROCK inhibitor treatment leads to cytoplasmic Yap localization in embryos.

### 2.2. Generating knock‐in (KI) reporter mouse lines by 2C-HR-CRISPR

The KI reporter mouse line was generated following our published protocol using 2C-HR-CRISPR on the CD1 background [11,12]. Briefly, Cas9 monomeric streptavidin (mSA) mRNAs were produced by *in vitro* transcription using the mMessage mMachine SP6 Kit (Thermo Scientific). PCR templates were generated by PCR reaction with biotinylated primers using high fidelity ClonAmp HiFi PCR mix (Takara Inc.). A mixture of Cas9-mSA mRNA (75 ng µl^−1^), sgRNA (50 ng µl^−1^) and biotinylated repair donor (20 ng µl^−1^) was microinjected into the cytoplasm of 2-cell stage mouse embryos and transferred the same day to pseudo-pregnant females. Founder pups were obtained from the pregnancies.

We established founder mice with the correct insertion at high efficiency (2/2 live born pups). A founder was outcrossed five generations to wild-type CD1 mice to breed out any potential off-target mutations introduced by CRISPR–Cas9 and then bred to homozygosity at N6 generation. The mouse line was then maintained by homozygous breeding. In the early generations of homozygous breeding, a cataract phenotype was observed in some mice. This phenotype was then removed by selectively breeding YAP-emiRFP670 homozygous reporter mice without such a phenotype. The homozygous mice are otherwise healthy and fertile without apparent phenotype.

For generating *Yap-emiRFP670/Cdx2-eGFP* embryos, the *Yap-emiRFP670* mouse line was bred to the *Cdx2-eGFP* mouse line to generate double homozygous mice [13]. Embryos were then collected from the double homozygous mouse line for imaging.

All animal work was carried out following the Canadian Council on Animal Care Guidelines for Use of Animals in Research and Laboratory Animal Care under protocols approved by the Centre for Phenogenomics Animal Care Committee (20–0026H).

### 2.3. Genotyping and genetic quality controls

Founder mice were genotyped by PCR amplification with primers spanning homology arms using the following primers: 5′ arm gtF: GTTCTAAGGTAGACACTGTGTGCTTCAGTT and 5′ arm gtR: TCATGTTCGCAGGTCAAGAGGTCA; 3′ arm gtF: CTGGTTGTCTGTCACCATTATCTGC and 3′ arm gtR: AACACCTGCAATTGCTCCAACC. Founder mice were outcrossed to CD1 mice to generate N1 mice. The N1 mice were genotyped by PCR. Additionally, genomic regions spanning the targeting cassette and 3′ and 5′ homology arms were Sanger-sequenced to validate correct targeting and insertion copy number was evaluated by droplet digital PCR (performed by the Centre for Applied Genomics at the Research Institute of The Hospital for Sick Children, Toronto). Heterozygous N1 mice have only one insertion copy, demonstrating single-copy insertion.

### 2.4. Embryo isolation, culture and treatments

Preimplantation embryos were isolated from superovulated females that were mated with males, both homozygous for the C-YAP reporter. Embryos were isolated from oviducts or uterus at appropriate stages for each experiment—E0.5 for zygote, E1.5 for 2-cell embryos, E2.5 for 8-cell embryos and E3.5 for blastocysts. Embryos were flushed with M2 medium. They were then cultured in small drops of KSOM-AA medium under mineral oil at 37°C, with 6% CO_2_ for specified times. For the Rock inhibitor treatment experiment, 8-cell stage embryos were cultured in KSOM-AA medium supplemented with 20 µM Y-27632.

### 2.5. Time lapse live imaging preimplantation embryos

For imaging the YAP dynamics during the formation of 16-cell embryos, embryos were flushed from the oviduct at 4-cell or 8-cell stage and cultured in a 3 µl drop of KSOM-AA medium under mineral oil on a MatTek glass-bottom dish. Live imaging was performed at 20 min frame^−1^ for 24–36 h. For the ROCKi inhibitor treatment, embryos were flushed from the oviduct at 8-cell stage and cultured in a 3 µl drop of KSOM-AA medium with 20 µM Y-27632 under mineral oil on a MatTek glass-bottom dish. Live imaging was performed every 90 min for 24 h.

### 2.6. dnLATS2 mRNA injection

Homozygous *Yap-emiRFP670* embryos were collected at 2-cell stage. mRNAs were microinjected into one of the two blastomeres as previously described [7,14]. For control experiments, embryos were injected with H2B-RFP mRNAs at 300 ng µl^−1^. Treated embryos were injected with dnLATS2 mRNA at 1000 ng µl^−1^ plus H2B-RFP mRNA at 300 ng µl^−1^. The embryos were then cultured to the early blastocyst stage for immunofluorescent analysis.

### 2.7. Whole-mount immunofluorescent staining of embryos

Whole-mount immunofluorescent staining of embryos was performed as previously described [15]. Briefly, embryos were fixed in 4% paraformaldehyde at room temperature for 15 min, washed once in PBS containing 0.1% Triton X-100 (PBS-T), permeabilized for 15 min in PBS 0.5% Triton X-100 and then blocked in PBS-T with 2% BSA (Sigma) and 5% normal donkey serum (Jackson ImmunoResearch Laboratories) at room temperature for 2 h, or overnight at 4°C. Primary and secondary antibodies were diluted in blocking solution, staining was performed at room temperature for approximately 2 h or overnight at 4°C. Washes after primary and secondary antibodies were done three times in PBS-T. Nuclear staining was performed using Hoechst 33258 (Thermo scientific) at a concentration of 10 µg ml^−1^ for 20 min at room temperature. Embryos were mounted in PBS in wells made with Secure Seal spacers (Molecular Probes, Thermo Fisher Scientific) and placed between two cover glasses for imaging. Primary antibodies: Goat anti-tdTomato (Biorbyt orb182397 Lot AR2150) at 1 : 200 dilution, Rabbit anti YAP (Cell Signalling Technology (D8H1X) XP Ref 11/2018 Lot4) at 1 : 100 dilution and Mouse anti Cdx2(Biogenex MU392A-UC Lot MU392A0714) at 1 : 100 dilution. Secondary antibodies all from Themo Scientific and at 1 : 400 dilution: Donkey-anti-goat IgG AF 546 (A11056 Lot 1714714), Donkey anti Rabbit IgG AF647(A31573 Lot 1693297) and Donkey anti mouse IgG AF488 (A21202 Lot 1741782).

### 2.8. Confocal imaging of preimplantation embryos

Both live and immunostained images of preimplantation embryos were acquired using a Zeiss Axiovert 200 inverted microscope equipped with a Hamamatsu C9100-13 EM-CCD camera, a Quorum spinning disk confocal scan head, and Volocity acquisition software v. 6.3.1. Single plane images or *Z*-stacks (at 1 µm intervals) were acquired with a 40*x* air (NA = 0.6) or a 20*x* air (NA = 0.7) objective. Images were analysed using Volocity software. Live imaging was performed in an environment controller (Chamlide, Live Cell Instrument, Seoul, South Korea) on the same microscope.

**Figure 2.**
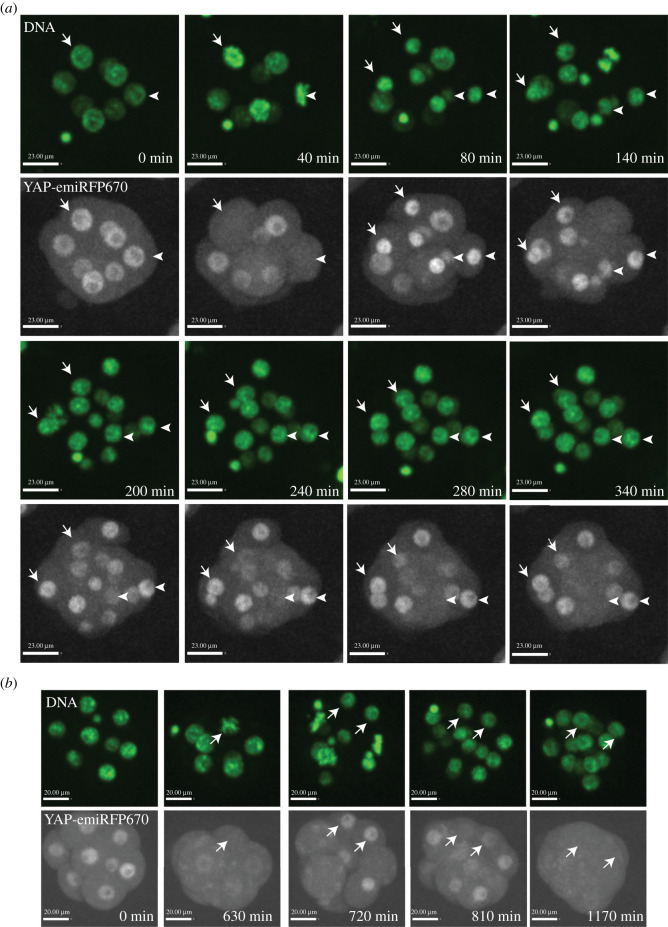
Dynamic YAP localization in 8–16-cell mouse embryos. (*a*) Snapshots from live imaging series (electronic supplementary material, movie S3) as examples of YAP behaviour in mitotic pairs. For the pair annotated by arrows, both cells presented nuclear YAP after the cell division at 80 min, and both of them located on the outside of the embryo and maintained nuclear YAP at 340 min. For the pair annotated by arrowheads, both cells presented nuclear YAP after the cell division at 80 min. Subsequently one of them was located on the outside of the embryo and maintained nuclear YAP while the other moved to the inside and presented cytoplasmic YAP at 340 min (quantitative data in Extended figure 3*b*–*d*). (*b*) Snapshots from live imaging series (electronic supplementary material, movie S4) with *Yap-emiRFP670* embryos treated with the ROCK inhibitor Y-27632. During the 8-cell stage, 0–630 min, ROCK inhibition resulted in primarily cytoplasmic localization of YAP. After cell division, as demonstrated by the cell pair marked by arrows at 720 min, all cells showed a transient nuclear localization of YAP. Then all cells gradually reversed to a cytoplasmic YAP localization status over time (810 min and 1170 min).

Time lapse imaging was performed on the same microscope equipped with an environment controller (Chamlide, Live Cell Instrument, Seoul, South Korea). Embryos were placed in a glass-bottom dish (MatTek) in KSOM-AA covered with mineral oil. A 20*x* air (NA = 0.7) objective lens was used. Images were acquired at 1–3 µm *Z* intervals with time lapse settings as indicated in the legend to figure 2*a*.

### 2.9. Image quantification analysis for preimplantation embryos

Preimplantation images were visualized using the Volocity 6.3 software (see https://www.perkinelmer.com/en-ca/lab-products-and-services/resources/whats-new-volocity-6-3.html). The live images of preimplantation embryos were traced manually by carefully inspecting the movie and tracking the cell nucleus marked by the live DNA dye and recording the presence or absence of the YAP-emiRFP670 reporter. For quantifying fluorescent intensity, images were exported as TIFF files and measured using the region of interest (ROI) function in ImageJ 1.53a software (see https://imagej.nih.gov/ij/notes.html). The average fluorescent intensity of each nuclear ROI was measured and subtracted against a general background fluorescent intensity in the corresponding image.

### 2.10. Lightsheet imaging of post-implantation embryos

Three-dimensional static live imaging was performed on a Zeiss Lightsheet Z.1 lightsheet microscope. Embryos were suspended in a solution of DMEM without phenol red containing 12.5% filtered rat serum and 1% low-melt agarose (Invitrogen) in a glass capillary tube. Once the agarose solidified, the capillary was submerged into an imaging chamber containing DMEM without phenol red, and the agarose plug was partially extruded from the glass capillary tube until the portion containing the embryo was completely outside of the capillary. The temperature of the imaging chamber was maintained at 37°C with 5% CO_2_. Images were acquired using a 20× objective with dual-side illumination in a multi-view mode (four evenly spaced views spanning 360° for E8.0 embryos imaging) or tile scanning mode (for E8.5 and E9.5 embryos imaging). The light sheet images were processed using Zen (Zeiss), Arivis Vision4D (Arivis), Imaris (Bitplane) and ImageJ.

Time lapse imaging was performed similarly to the static live imaging with the following modifications: (i) 2% fluorescent beads (1 : 1000, diameter: 2 µm; Sigma-Aldrich) were added to the low melting point agarose for drift compensation. (ii) Images were acquired for 3 h with 5 min intervals.

### 2.11. *In vivo* drift-compensated cell tracking and mean squared displacement calculation

The light sheet time lapse image was first rendered in Imaris (Bitplane). Nuclear YAP positive cells were determined by mean thresholding of fluorescence intensity. For each embryo, the cut-off intensity was set to be 50% of its maximum intensity. Small bright spots of YAP (diameter less than 7 µm) due to local chromatin condensation were excluded from nuclear YAP positive cell identification. The positions of nuclear YAP positive cells were tracked over time using an autoregressive motion algorithm. The tracking data were then imported into MATLAB (MathWorks) for drift compensation using a program reported before [16].

Mean squared displacement (MSD) is an unbiased metric to evaluate cell migration [17]. For an arbitrary trajectory, the MSD and time delay follows a power-law relation, with power of 0 representing the random walk motion, and power of 2 representing the straight-line motion. On a log–log plot, these two cases translate into a line with slope 0 (i.e. a horizontal line) for the random walk motion, and a line of slope 2 for the straight-line motion. The mean MSDs of nuclear YAP positive cells’ migration trajectories are characterized by a line of slope 1.433 as shown in figure 4*c,* which deviates significantly from the random walk motion suggesting a persistent cell migration [18].

### 2.12. Statistical analysis

Statistics on numerical data were performed using the Prism 9 software (GraphPad Software, LLC). For intergroup comparison, the data were first subjected to the D'Agostino and Pearson normality test. Datasets that conform to a normal distribution were then subjected to the unpaired Student's-*t* test, and the ones that did not conform to a normal distribution were then subjected to the Mann–Whitney test. Two-tailed analysis were used in all cases. Exact *p*-values were presented in the figures. For the one-group *t*-test for the proportion of relative position of cell division pairs at the 16-cell stage, we tested against a null hypothesis that the proportion = 50%, which resulted in a *p*-value of 0.0381.

## 3. Results and discussion

We set out to engineer a knock-in (KI) fusion reporter of *Yap* in mice (Extended figure 1 in the electronic supplementary material). The function of mammalian YAP seems to be easily disrupted by fusion tags. We tested different combinations of tagging position and linker sequences and finally successfully generated a healthy reporter mouse line by C-terminal tagging with a long (30 amino acid) flexible linker (Extended figure 1*a*). Other designs, such as N-terminal tagging with the same linker led to embryo death at embryonic days 8.5 (E8.5), similar to *Yap* knockout embryos, suggesting functional interference [19]. To allow good light penetration for imaging deep tissue layers in post-implantation mammalian embryos and other tissues, we chose to use a bright near-infrared (NIR) fluorescent protein (FP)-enhanced miRFP670 (emiRFP670), as the fluorescent indicator [20]. We performed extensive quality control to confirm single-copy insertion of the fusion reporter with the correct sequence, as detailed in the methods section and extended figure 1*b* and our published protocol [11,21]. The homozygous mice are healthy and fertile. The mouse line was maintained in the homozygous state after outcrossing for five generations to ensure no carry-over of any possible off-target alterations.

We validated the reporter in mouse preimplantation embryos where the YAP distribution and its responses to various interventions are well characterized. The nuclear/cytoplasmic distribution of YAP from 8-cell stage onward is well established from previous research: At 8-cell stage, all blastomeres have nuclear localized YAP. From 16-cell stage to early mid blastocyst stage (E3.5), nuclear YAP is restricted to the outer cells that will give rise to the trophectoderm [7]. By the expanded blastocyst stage (E4.5), some epiblast cells will start to present nuclear YAP status [22]. Our live imaging reproduced this pattern (figure 1*a*). Before the 8-cell stage, the localization of YAP distribution is more debatable. Our live imaging showed that YAP was evenly distributed in the cytoplasm and nucleus of blastomeres until the late 4-cell stage, at which point YAP begins to be more restricted to the nucleus (figure 1*a*). We further validated the colocalization of the YAP-emiRFP670 reporter with the endogenous YAP protein and with the expression of CDX2 protein in the trophectoderm (TE) of blastocysts by immunofluorescence (figure 1*b*).

We then investigated whether the YAP-emiRFP670 reporter protein can respond appropriately to exogenous signals. In mouse early embryos, YAP localization is controlled by HIPPO, polarity signalling pathways and cortical tension [23–28]. From the HIPPO signalling pathway, LATS1/2 kinase is responsible for the phosphorylation of YAP, which leads to its sequestration in the cytoplasm [7]. Overexpressing a kinase-dead LAT kinase (dnLATS2) can promote the nuclear localization of YAP and the expression of CDX2 even in inside cells [7]. This result was replicated when we injected a dnLATS2 mRNA into homozygous *Yap-emiRFP670* embryos (figure 1*c*). As for polarity and cortical tension, it has been shown that the inhibition of the Rho-associated protein kinase (ROCK) kinase using a small molecular inhibitor Y27632 resulted in cytoplasmic YAP localization across all cells of the embryo, and as a result, failure to establish the TE [29]. We cultured *Yap-emiRFP670* reporter embryos from 8- to 16-cell stages in Y27632 and live imaged them. As shown in figure 1*d* and electronic supplementary material, movie S1, ROCK inhibition indeed resulted in a cytoplasmic YAP localization in all cells (figure 1*d*).

We then used the validated reporter mouse line to analyze the behaviour of YAP during embryonic development in real-time. A key event that YAP regulates during preimplantation development is the initiation of inner cell mass (ICM)-TE segregation at the 16-cell stage. When 8-cell embryos transition to 16-cell embryos through cell division, YAP becomes localized in the nucleus of blastomeres located on the surface of the embryos (outside cells) and excluded from the nucleus in inside cells [7]. The presence of nuclear YAP drives the expression of the TE-specific transcription factor CDX2 in outside cells, initiating TE lineage differentiation. By contrast, the enclosed blastomeres with cytoplasmic localized YAP initiate *Sox2* expression and ICM differentiation [30]. However, the exact process that leads up to this asymmetric YAP signalling status is still debatable. Some models suggest a fixed determination of YAP localization at the time of generation of inside and outside 16-cell blastomeres, while other recent studies suggest a more dynamic process [31,32]. Direct observation of the YAP dynamics through the 8–16-cell stage is the best way to resolve these models.

We time lapse imaged homozygous YAP-emiRFP670 reporter embryos every 20 min at the transition between the 8-cell to 16-cell stage for a period of 20 h. Cell nuclei were marked by a live DNA dye (electronic supplementary material, movies S2 and S3, with additional movie from 4-cell stage-electronic supplementary material, movie S4). To rule out the possibility of the imaging process affecting normal development, we further cultured the embryos to E4.5 and validated a high blastocyst formation rate (8/9) (Extended figure 2*a* in the electronic supplementary material). The movies revealed profound dynamic movements of blastomeres in 8–16-cell stage embryos. Many cells changed their relative outside–inside position from where they were localized right after the cell division that generates them (electronic supplementary material, movies S2 and S3). We discovered an intriguing mitotic reset behaviour pattern of YAP during this transition through closer inspection and tracking cells from the movies. When an 8-cell blastomere divides to form two daughter cells—16-cell blastomeres—both always show nuclear localization of YAP, regardless of the direction of the cell division axis (figure 2*a*). It took roughly 100–300 min for the two daughter cells to adopt their final position in the embryo (figure 2*a*). The final position that a cell adopted determined its YAP distribution—when a cell adopted an outside position, it presented nuclear YAP, whereas when a cell adopted an inside position, it presented cytoplasmic YAP (examples in figure 2*a* and quantifications in Extended figure 2*b*,*c*). There was an almost equal chance for two 16-cell blastomeres from a single 8-cell blastomere to adopt one of the two relative positions—outside–outside or outside–inside (Extended figure 2*d*). Previous reports have suggested that, rather than oriented cell division, a significant proportion of inside cells at the 16-cell stage are internalized by cell movement, or asymmetrical constriction of the apical domains [25,32]. Both of these scenarios are consistent with our observations. However, because we did not include a reporter of the apical domain, our imaging studies cannot distinguish these two scenarios and further studies will be needed. The YAP-emiRFP670 reporter allowed us to relate this dynamic cell movement to a dynamic regulation of YAP signalling. Interestingly, the movie of embryos treated with ROCK inhibitor (electronic supplementary material, movies S1 and S5 and figure 2*b*) showed that although ROCKi eventually inhibited YAP nuclear localization in all 16-cell blastomeres, the initial YAP nuclear localization right after cell division was not affected, suggesting a differential involvement of ROCK-related processes—such as polarity and mechanical tension—in regulating these two distinct YAP nuclear localization processes. How this dynamic behaviour is controlled remains an open question, which can now be addressed by live imaging the YAP reporter model in combination with additional KI reporters tracking polarity and mechanical tension.

**Figure 3.**
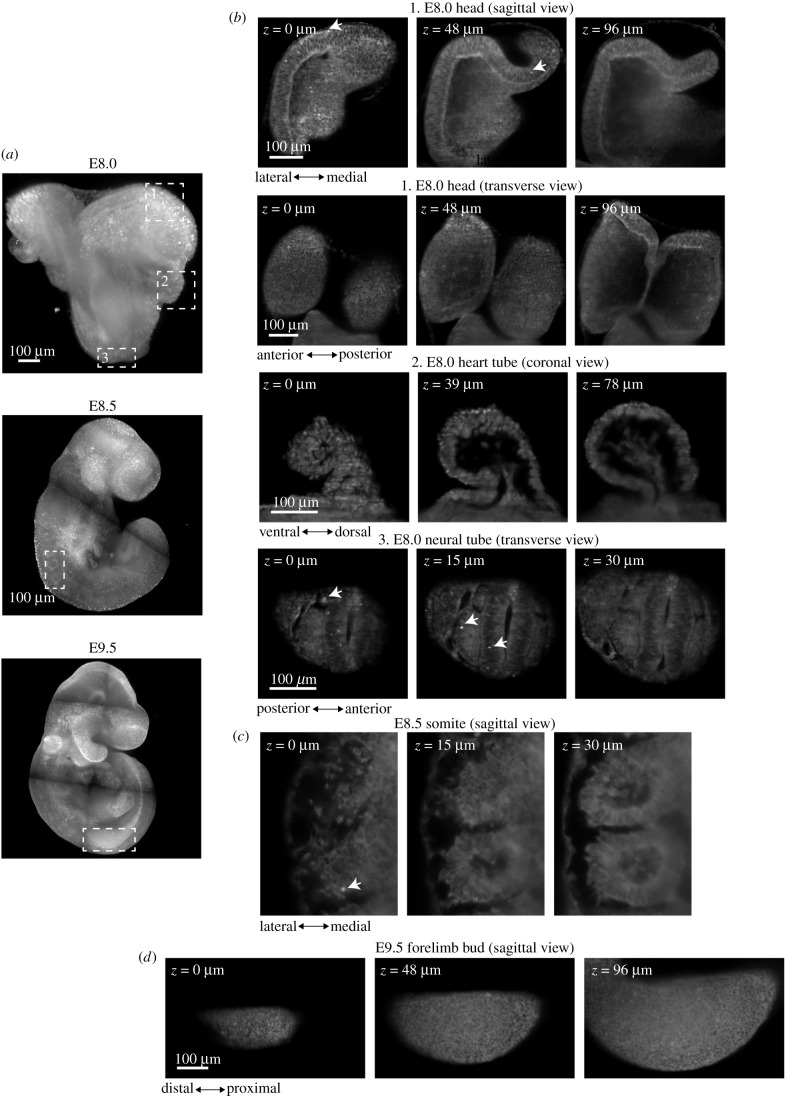
Live imaging of Yap in early organogenesis-stage embryos. (*a*) Tile scan images of C-YAP embryos at three developmental stages: E8.0 (before turning), E8.5 (after turning) and E9.5. (*b*) Zoom-in views of square-marked regions in the E8.0 embryo in figure 3*a*: 1. head fold, 2. heart tube and 3. neural tube. (*c*) Zoom-in views of the somite region in the E8.5 embryo (square in image). (*d*) Enlarged views of the limb bud region in the E9.5 embryo (square in image). Arrows in (*b*,*c*) mark examples of cells with strong nuclear Yap.

Preimplantation embryos are small and transparent. To demonstrate the broader application of the YAP reporter in more challenging samples, we live imaged the YAP-emiRFP670 embryos at E8.0 (before turning; electronic supplementary material, movie S6), E8.5 (after turning, electronic supplementary material, movie S7) and E9.5 (electronic supplementary material, movie S8) using light sheet imaging technology. We achieved high-resolution imaging of multiple tissues including those located in deep tissue layers (up to 200 µm), such as the heart tube (figure 3 and *Z*-stack in electronic supplementary material, movie S9). In most regions, tissues consisted primarily of cells with cytoplasmic YAP, with only small populations of cells with strong nuclear signals (figure 3*b*–*d*, examples marked by arrows). In the heart tube, on the other hand, most cells showed nuclear YAP, which may be consistent with the mechanical load these cells are subjected to and suggests a crucial role for YAP in heart development [33,34]. All the raw imaging data of the sub-cellular distribution of YAP in post-implantation embryos will be deposited on open access databases and will serve as a rich resource for studying YAP signalling in mouse embryos.

**Figure 4.**
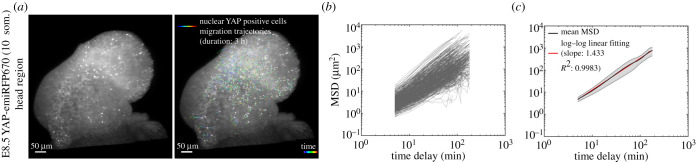
Migrating cells (*a*). Migration trajectories of nuclear YAP positive cells within the head region of a E8.5 YAP-emiRFP670 (10 som.). Left image shows an embryo with bright nuclear YAP cells at the beginning of the time lapse imaging. Right image depicts the migration tracks of these cells over 3 h. Colourmap represents the time points of individual cell migration trajectories over 3 h imaging with blue and red being 0 and 3 h, respectively. (*b*) MSD (mean squared displacement) of nuclear YAP positive cells’ migration trajectories shown in A (520 tracks). (*c*) Mean MSD (black line) of nuclear YAP positive cells’ migration trajectories at E8.5 (10–11 som.) head regions (1962 tracks from three embryos). Log–log linear fitting (red line) yields a slope of 1.433 indicating a persistent cell migration motion (Methods). Grey colour marks the standard error of mean.

To reveal the dynamic behaviour of cells with active YAP signalling, we time lapse imaged E8.5 embryos every 5 min for 3 h (electronic supplementary material, movie S10). This movie revealed a population of cells with strong nuclear YAP signal migrating within the head region (figure 4*a* and electronic supplementary material, movie S10). We conducted tracking of the time-dependent motion of these nuclear YAP cells [16]. The persistence of these cellular motions was tested using MSD from 1962 tracks from three embryos and showed a strong deviation from a random walk model, suggesting persistent cell migration (figure 4*b*,*c*). The identity of these cells and the mechanisms by which YAP regulate directional cell migration in embryos warrant further investigation. These data demonstrate that the C-YAP reporter can have broad imaging applications in challenging samples such as late-stage embryos and tissues.

### 3.1. Open up

The YAP-emiRFP670 mouse line will open up broad opportunities in biomedical research. For example, existing studies in early embryos suggest that at the very early totipotent stages of development (zygote to pre-compaction 8-cell stage), YAP primarily serves to open up the zygotic genome [35,36], and then it transforms to a lineage determinant around the 16-cell stage [7]. We conducted a time lapse imaging series with YAP-emiRFP670 and a trophectoderm reporter Cdx2-eGFP (electronic supplementary material, movies S11–S13). The movie revealed very little correlation between the YAP nuclear localization and Cdx2 expression up to the late 16-cell stage. In addition, although ROCK inhibitor treatment caused the cytoplasmic localization of YAP at late 16-cell stage, the CDX2-eGFP persisted (electronic supplementary material, movie S14). These data suggested a more complex relationship between the nuclear YAP activity and the expression of TE markers such as CDX2. Live imaging could help define the precise timeline over which YAP acts as a lineage determinant and lead to further understanding of the transition of YAP functions in early embryos. In addition, the deep imaging capability provided by this reporter can illuminate previously unknown YAP activity status in embryos or other three-dimensional model systems such as organoids. Our validation of a knock-in fusion design that maintains the normal functions of endogenous YAP can also serve as the ground-plan for developing other powerful genetic tools such as degrons and optogenetic tools for further functional interrogation of YAP signalling *in vivo*.

In summary, we present the first KI fusion reporter mouse model to enable the readout of YAP nuclear–cytoplasmic localization by live imaging. Using this line, we reveal new aspects of dynamic YAP behaviour in preimplantation mouse embryos and the capacity to live-image post-implantation embryos with penetration at depths of up to 200 µm. This live imaging YAP reporter can be combined with other appropriate reporters to study multiple developmental and disease processes.

## Data accessibility

The raw data that support the findings of this study have been uploaded to: https://figshare.com/articles/dataset/Yap_live_imaging/19319885.
